# An Essay on the Most Important Diseases of Women

**Published:** 1839-04-01

**Authors:** 


### Essays on the most important Diseases of Women. By *Robert Ferguson*, M.D. Part I. Puerperal Fever. 1839.

We shall be much deceived if this work, even if no other parts succeed it, do not lay a lasting foundation for its author's reputation. Dr. Ferguson goes to work in the true way. He deduces legitimate consequences from authentic facts. His materials are drawn, without exception, from the records of the General Lying-in Hospital, during a period of twelve years, and amounting to 204 cases of a disease which causes seven-eighths of the mortality in child-bed. These cases are not narrated by the author, and dressed up, as too frequently occurs, according to the fancy, or interest, or vanity of the recorder. They are the bed-side notes of the ablest pupils, inscribed day by day, and left for the inspection of all. Those who are employed in the wide field of actual practice in the sick chamber, rather than in the library of the briefless critic, know well the difficulties which beset the physician or surgeon at the bed-side of puerperal fever. No subject has given rise to more clashing and perplexing incongruities both of theory and practice, than the one under discussion. Tonellé (one of the latest and best writers) observes that, "a comparison of its phenomena (puerperal fever) as seen in various epidemics, and a fusion of separate opinions into one collective result, will alone permit us to understand and to alleviate this formidable disease." Dr. F. has set this standard before him, and has endeavoured, to the best of his abilities, to compare his own experience with that of others. We shall proceed at once to a close and copious analysis of this important Essay—a somewhat difficult task, since there is scarcely a word superfluous or that could be spared, from beginning to end. The language is elegant, terse, and perspicuous, without the slightest attempt at meretricious ornament. It is, indeed, a perfect model for medical literature. It is the Celsus of the 19th century.

Chapter the First exhibits the various forms of puerperal fever. These are four—all distinct in their general characters, and requiring different treatment. The first is the *peritoneal* form—the intensity of the malady falling on that tissue, and lingering longest there. The second form consists of *general fever*, with much disorder of abdominal viscera—the symptoms being often *remittent*, sometimes even *intermittent*. In the third form, the *brain and nervous system* suffer most—whilst the fourth form seems to be a complication of the other three—with this dangerous feature in addition—a tendency to effusion in any or every tissue—of blood, serum, pus, and lymph. The last kind of effusion is the most common, and the most dangerous—especially in crowded hospitals, and in the abodes of misery and filth.

"These four forms—the peritoneal, the gastro-enteric, the nervous, and the complicated, spring, as I shall endeavour to shew, from one source and cause. There is no trenchant limit which bounds them in nature; and in every epidemic which I have witnessed, the characteristics peculiar to any one are readily assumed by another. In the peritoneal form, the peritonitis ceases, and the patient succumbs under fever and diarrhœa—or under a complication of local effusions and phlegmasiæ. In the gastro-enteric, sudden peritonitis will supervene, and carry off the patient in a few hours. Such also may occur in the third or nervous form, or it may (though it rarely does) terminate by a lengthened fever, or by deposit in the great cavities, or in the joints, the muscles or the eye-balls. It is the last form alone which is invariable, save in intensity. It may be looked on as a summary of the others, while these may be considered as fragments of it." 2.

The one or other of these forms generally gives the character to an epidemic—swallowing up, as it were, the other forms—hence the confusion and discrepancies of authors respecting the symptoms and treatment of the malady. The variations in intensity, as well as in form, have proved another fertile source of contrariety of opinion amongst observers. After animadverting on the various classifications of authors, Dr. F. proceeds to the—

1. PERITONEAL FORM.—This is characterized by abdominal pain—of two kinds—the one durable and dangerous—the other transient, and easily removed, if uncomplicated with other disease. The difference between plus and minus in the ratio symptomatum is not very easily estimated.

"Of two patients attacked by abdominal pain, it will in the majority of cases, and at the commencement of an epidemic, be very difficult to ascertain which is the slighter and which the severer malady. In both, the intensity of anguish, the seat of the pain included between the pubes and a line drawn from the superior crest of one ilium to that of the other ;—the precursory rigor followed by the hot fit ;—the time of attack, from the first to the fifth day after parturition, are all the same, and neither the pulse, nor the degree of fever distinguish the one from the other. The action of remedies, however, shews their distinctive characters. The transitory form being readily relieved by such agents as lull pain, while the other requires such as are used to quell pure inflammatory action. The transient abdominal pain passes into the second, or permanent kind; but in some epidemics it forms the principal character of the common malady, and I have never seen one, in which some of these cases did not occur. In the year 1827, and a part of 1828, this form of malady was very frequent, and I had repeated opportunities of pointing it out to the pupils of the General Lying-in Hospital, with whom it obtained the name of false peritonitis. One of these, my friend Mr. Hingeston, subsequently published cases which he saw during the period of his residence at the hospital, which possess the double advantage of being well reported, and of authenticating the existence of a form of malady which has been denied by some." 11.

In the epidemic of 1827-8, this form of puerperal affection was very prevalent along the banks of the Thames, and Dr. F. was so worn out with incessant calls, that he directed the matron to send to each applicant two ten-grain powders of the pulv. ipecac. comp., one to be taken immediately, and the other four hours afterwards. If the peritoneal pain continued after the second dose, then the Doctor was ready to attend. After this regulation he was relieved from four-fifths of his visitations.

"As to the nature of this form of puerperal fever, Mr. Hingeston looks on it as nervous, only in the sense in which a nervous impression precedes every inflammation ; the peculiarity being its permanence in this early stage. Mr. Griffin regards it as nervous pain, dependent on spinal irritation ; M. Tonellé, as connected with the circulation of pus in the veins.

I believe these opinions are not really different, except in each being only a part of the truth. M. Tonellé, I think, and shall prove hereafter, would have given the real cause had he generalized his proposition into a vitiated state of the blood, instead of limiting such vitiation to one cause, namely, the circulation of pus. In many instances we have the genuine marks of vitiated fluids, without being able to detect a particle of pus in them. Le Gallois mixed pus with blood, while it flowed from the arm, but could not, subsequently, detect in the compound any of the matter; hence, pus may exist in the circulation without our being able to discover it. But I shall, hereafter, show that other products of inflammation will vitiate the blood." 18.

Our author considers that the treatment of this incipient disease is not a matter of indifference. He believes that a large bleeding in such a stage would change its transitory into a permanent character, and render it a formidable malady. The diagnosis, however, is allowed to be difficult.

"If the pain be very sudden in its onset; if there be *intermission,* we may fairly expect relief from opiates. If there be *remission,* well marked, we may resort, with much hope, to the same treatment.

If the pain be constant, we may, under the following limitation resort to opiates in the first instance, namely; 'if our previous experience of the reigning epidemic has proved that the peritoneal pain has in other cases easily shifted.' Hence the *nature* of the epidemic is to be studied, and used as a guide.

Lastly, we may, in doubtful cases, resort to the opiate treatment, and, should it not have produced, in four hours and a-half, after two 10 grain doses of Dover's powder, a marked amelioration, we ought, forthwith, to resort to local or general depletion.

The routine of the hospital practice in cases of abdominal pain has been this. A hot linseed-meal poultice, immediately on complaint of pain—and this as it induces perspiration, is very often alone sufficient; then, an opiate with or without an aperient; and in the event of no amendment within four or six hours after the attack, a change of treatment according to the nature and grade of the disease." 21.

The second or permanent form requires local or general depletion of an energetic character. It has four stages—the first an ague fit—the second marked by severe abdominal pain, fever, hard pulse, restrained breathing. The third stage is that of effusion, marked by *apparent* amelioration. The fourth is collapse, with change of countenance, laborious respiration, cool surface, swollen abdomen, pulse rapid—mind usually clear. Purely peritoneal inflammation, however, is comparatively rare in puerperal women. Other organs are generally implicated.

2. Second Form—Gastro-enteric Irritation.—This form assumes the general character of mild typhus fever, accompanied by intestinal irritation. There is no peritoneal affection, or it is very slight, being lost in the general disturbance. It rarely lasts less than seven or more than twenty days. It is ushered in by rigors and then reaction.

"From the very onset of the disease, there is some marked irritation of the mucous membrane of the intestinal canal—either vomiting, nausea, or diarrhœa, in which the evacuations exhibit every kind and grade of vitiated secretion, varying in colour, consistence, offensiveness, and frequency. The tongue, at first loaded and white, soon becomes preternaturally red; as in those affected by chronic dysentery. The skin is dry, hot, and of a dusky yellow hue; the mind is unsettled, without being absolutely delirious, and the impressions on the eyes are unwontedly vivid; the debility is extreme, and the limbs very tremulous. There is a very marked remission during the day, and an equally marked exacerbation towards evening, and night, when the dreams of a troubled sleep are so vivid as to impress the mind with all the distinctness of reality. Even during waking, the patient sees objects flitting constantly before her eyes. In some instances there is watchfulness and mild delirium during the night, with perfect mental collectedness during the day. This grade and form of puerperal fever rarely or never terminates fatally without first becoming complicated with some acute inflammation of an important organ, as the peritoneum, or the thoracic viscera; or some deposition in the joints or limbs, followed by colliquative diarrhœa. Hence, the lesions found after death are chiefly of the organs last attacked, while the primary affection of the intestinal canal, is scarcely marked by any structural disorganisation. The uterus, however, will, as usual, be found either much congested and large, or its veins and lymphatics containing pus, or its inner mucous surface superficially softened. When there is extensive softening of this viscus, all the above characteristics of this form of puerperal disease are merged in the broad and very obvious signs of an overwhelming, and from the very first, a fatal typhus. There is no peritoneal pain, but merely very deep-seated and obtuse sensibility to firm pressure. The mind and body are equally prostrate and enfeebled. The skin is of a deep-brown colour. The pulse small, weak, and very rapid. The abdomen soon becomes tympanitic, and the whole intestinal canal seems filled with dark fluid, which is vomited without effort in large gushes, or passed in unrestrainable diarrhœa. The less formidable of these two grades seems to have given the character to an epidemic, which was described by Dr. Butter in 1775, under the term of remittent puerperal fever." 24.

3. Third, or Nervous Form.—This, in a pure and isolated form is very rare, comparatively, though it is not unusual for it to set in, and, for a time, interrupt the course and character of some of the other forms of the disease. It is distinguished by terror of mind, coldness of skin, much agitation, and sense of impending death, faintness and prostration.

"Those in whom the nervous character is the sole, or at least the most prominent part of the puerperal fever, exhibit all its symptoms in all their irregularity and inconstancy; there is painful and sudden abdominal tenderness which subsides with extreme rapidity; there is a rapid pulse, great restlessness, and mental uncertainty and agitation, together with shifting functional disturbance of various organs; sighings, tremors and cramps; sudden and death-like sinking, and as sudden re-appearance of strength; with these, there are nevertheless, from the beginning of the attack, unequivocal marks of deep injury to the nervous system. The faculties and feelings are strangely disturbed, and the terror the patient expresses, or the furious delirium which often ushers in the attack, soon gives way to fatal coma, or sudden syncope." 26.

4. Fourth, or Complicated Form.—This is an appalling malady, being the simultaneous or rapidly successive attacks of various organs and tissues of the body. It begins from the first to the third day, with shivering, often followed by abdominal pain. The debility quickly amounts to prostration. The mind is calm, and unconscious of danger. The pulse is rapid, the skin dusky, with red patches on the cheek, the eye glassy, with lead-coloured lids. The abdominal pain sometimes last during the brief tragedy—sometimes subsides soon. The intestinal canal is usually the first to suffer in the list of complications—dysentery or diarrhœa, with tormina and bloody stools, taking place—terminating, at last, in coffee-coloured evacuations. The lungs are generally affected, functionally or organically. There is loud and incessant sighing—or short interrupted breathing—or pneumonia. The pleura is often attacked, and effusions take place into the cavity of the chest. Our author has seen gangrene of the œsophagus, and even of the lungs themselves, perforations of the stomach and intestines, lesions of the heart and its coverings, &c.

"In some instances the skin retains its natural colour, while in others the portion over the painful spot is red, tumefied, and very hot. Either this state gradually subsides, or a soft quaggy feel is communicated to the pressing finger, and then an effusion of pus, serum, or blood, or a mixture of all, will have taken place; after which there is usually complete relief to the limb, and a subsidence of febrile and other symptoms; nevertheless, these formations, regarded as critical by most observers, do not save the patient from dying of colliquative diarrhœa and perspirations, with their attendant exhaustion, except in the milder cases. Whenever these abscesses supervene, the result is very doubtful. When the effusion consists of pus, it is not contained in cysts, but lines the sheaths of the tendons, or is smeared over the muscular surfaces, or infiltrated between the fibres. The muscles of the back of the fore-arm, and those of the calves of the legs near a joint, are most frequently attacked." 28.

The joints are nearly as often the seat of disease as the muscles, and containing the same depositions. Dr. F. has never seen the osseous system attacked. Of the connexion between erysipelas and puerperal fever, Dr. F. may say that the two maladies are generally coexistent in his hospital. Gordon and Hey observed the same coincidence. The intensity of the disease differs in different epidemics.

"As to the organs attacked, taken isolatedly, my experience proves to me,—1. That each organ may undergo every grade of disease, from simple irritation, which will not proceed further, to total destruction and softening of its tissues. 2. That when many organs are attacked at once, or consecutively, they are not wrought up to one uniform pitch of malady; that while one is simply irritated, another is gangrenous. Thus I have seen co-existent, inflammation of the peritoneum, with painless and sudden perforation of the œsophagus. What are our indications for such a disease, and what our hopes of cure?" 35.

### Chap. II.—Morbid Anatomy.

The peritoneum, though the seat of pain during life, is often found pale on dissection. A certain degree of vascularity, or even inflammation, may, as in scarlatina and erysipelas, leave no trace of its existence, post-mortem. If the pain, however, has been of long duration, and intense, there will be more or less of redness, with effusions of serum, lymph, blood, pus, or coagulable lymph. The uterine peritoneum, and the ligamenta lata, are more frequently the seats of the phlogosis than other parts. Few changes are discovered in the mucous membrane of the intestinal canal. When affected there are either inflamed patches—softening and perforation—or ulceration. The stomach is often softened, and sometimes perforated. The intestines are usually distended with air, and containing large quantities of a brownish fluid. The liver is often affected. Its peritoneal surface being not unfrequently coated with a layer of lymph, and its substance distended, and softened. Abscesses are rare. The kidneys present traces of inflammation, with depositions of pus, flakes of lymph, &c.

*Uterus and Appendages.*—"The peritoneal coat is very frequently injected, covered with lymph, or raised by a subjacent layer of pus or blood, or both. Its substance is soft and flabby, and its contractile powers so thoroughly suspended, as to permit no diminution of its volume. It is as large ten days after delivery, as it was immediately after the expulsion of the placenta. Small abscesses are found occupying various depths of the uterine walls. There are patches of thoroughly dissolved uterine matter, the softening almost always commencing in the inner surface of this viscus, and sinking towards its peritoneal coat.

This softening belongs to that class of lesions which cause perforations in the stomach, and dissolutions of the muscles, so common in this malady.

The inner surface of the uterus is often smeared with a thick layer of gelatinous blood, underneath which, patches of reticulated lymph, tinged greenish brown, or modena colour, are found. Cruvelhier, Dugés, Seiler, have all looked on this layer as a false membrane, and not the remains of the decidua. I have examined the uterus to verify this opinion, and I am, on the whole, satisfied of its correctness." 38.

The chest, the vascular system, especially the veins, shew traces of the ravages of this terrible disease.

"The uterine veins, forming a mesh-work, like the structure of the corpora cavernosa penis, are chiefly affected. The lining membrane is very often quite pale, though covered with false membrane or with pus. Their coats are thickened and their cavities obliterated, or contracted from interval to interval, when the disease extends beyond the uterine substance. When the neighbouring veins are affected, the adjacent cellular membrane is hardened or infiltrated, or forms a bed for purulent matter. The uterine veins are often found perfectly healthy, when the spermatic or renal, and still more distant veins are thoroughly disorganized. Besides the presence of pus or lymph in the veins, gritty and grey, or light brown coagula are found. The mass of blood not unfrequently retains its fluidity after death.

In a certain number of cases no lesion can be discovered in the vein, but the presence of some unnatural fluid. It is disputed whether it is absorbed or the product of venous inflammation. It is of little moment which of the two opinions be adopted; the disease depends not on how the matter is produced, but whether it enters the circulation. Whether this be by absorption or by inflammation, puerperal fever is the result. The prevailing opinion of secondary abscesses being, not metastases, but new inflammations, is, I believe, the true one.

In the ligamenta lata the presence of pus may be almost always detected: it is usually more frequently found in the lymphatics than in the veins,—and, Cruvelhier is of opinion, that phlebitis is rare, in comparison with pus in the lymphatics. It is collected in small pouches, which give the lymphatic a beaded appearance, and render it readily traceable." 40.

Purulent depôts are found in the joints, muscles, and other structures of the limbs, and the body is more rapidly decomposed after death, than in other diseases.

Such is a succinct history of the ravages produced by puerperal fever. The dissections which our author has made, are sufficient to establish the existence of all the lesions enumerated, but not their relative frequency, which can only be done in very large hospitals, as those of Paris.

### Chap. III.—Nature of Puerperal Fever.

Hitherto we have been sailing on velvet. It required but a sharp eye, industrious fingers, and attentive observation, to chronicle the symptoms during life, and the morbid lesions found on dissection. But when we come to discuss the *nature* of puerperal, or indeed any fever, we must take a higher flight. "Paulo majora canamus." Our readers will, we think, have guessed by what has already transpired at the theory to which our author is coming.

"The three following propositions embody my views of the source and nature of puerperal fever.

I. The phenomena of puerperal fever originate in a vitiation of the fluids.

II. The causes which are capable of vitiating the fluids are particularly rife after child-birth.

III. The various forms of puerperal fever depend on this one cause, and may readily be deduced from it.

By the first proposition, I shall endeavour to shew that the cause assigned will account for the phenomena of puerperal fever. By the second, I shall prove that the assigned cause really exists; and by the third, I shall endeavour to trace the various forms of puerperal fever to the one source from whence all of them proceed." 53.

First Proposition.

The blood may be vitiated by the direct artificial introduction of noxious substances into the circulation—or by certain diseases, as scurvy, purpura, jaundice, &c. The results are, a tendency of the blood to escape in a hæmorrhagic form, or mixed with mucus or serum—or in the shape of various local affections of different parts of the body. When vitiating agents are introduced artificially, they generally shew their effects most in the neighbourhood of the point of introduction. Our author then goes on to analyze the experiments of Gaspard, and Cruvelhier, which we need not advert to here, as they are sufficiently known to our readers.

"From these six experiments of Gaspard, we may conclude that the vitiation of the fluids will produce general fever, with local irritation or inflammation of different organs at the same time; that in the first four this fever was accompanied with gastro-enteric disorder; in the other two, by general functional disturbance, resembling the nervous form of puerperal fever." 58.

Various other experiments of Gaspard and Cruvelhier are adduced, and commented on by our author.

"From these and other experiments, Cruvelhier concluded, by believing that in inflammation the veins are chiefly affected; that the redness is venous, that the pus effused is from a simple rupture of veinules; that in inflammations of degenerate tissues, it is the veins which are developed, as in soft cancer and cephaloid tumor. Without entering into this hypothesis, we see that there are two sources from which the blood may be vitiated—either by the primary injection, or absorption of injurious substances; or by direct injury to the solid coats of the veins, which in consequence inflame and *secrete a fluid or fluids, which mingling with the torrent of the circulation,* act as if they had been primarily injected." 65.

"Gaspard's and Cruvelhier's experiments prove, that many substances will produce the same fatal effects, and by the same disorganising process; that mercury, thick unctuous substances, acrid fluids, gritty powders, and bits of stick, when placed within the vessels, all produce the same essential train of symptoms; while saliva, milk, urine, bile, cause little disturbance. Many of the deleterious substances produce no action on the coats of the large vein, and yet the symptoms resulting are precisely those which a wound and inflammation of the vessel will give rise to,

In the first case we are certain the cause of death is to be sought in the action of these substances, not on the injured vein, but on the blood. And in the second, we are equally certain that those very fluids are poured out by the inflammation of the vein, which if injected into an uninflamed vein of a healthy animal, would produce death. We should conclude, therefore, that it is the vitiation of the blood, and not the inflammation of the coats of the vein, which produces the disease.

It may be said, that though it be granted that the phenomena consecutive of artificial vitiation of the blood strongly resemble those of puerperal fever, yet that this identity of cause may after all be only a probable surmise, and that no certain conclusion ought to be drawn, unless it could be proved that putrid substances absorbed by the uterine vessels produce puerperal fever.

Such an experiment has unfortunately too often been made; and Gordon, Campbell, and Kirkland, have most distinctly acknowledged, that retained and putrid placenta or coagula will produce a genuine puerperal fever, not to be distinguished from that they have each described." 67.

Cases are related from Gordon and others in illustration, and then Dr. F. proceeds to—

PROPOSITION II.

The object is to prove that the causes of vitiated blood are peculiarly rife in the puerperal state. Thus are the vessels mechanically injured? Are they in contact with any noxious matters? The uterus, he avers, after childbirth combines both these conditions. All the uterine veins and arteries have been torn from the placenta, and they form a part of a large wound. They are bathed in all the secretions that take place while this wound is healing. "In this respect the uterus presents an exact analogy to the surface of an amputated stump; and it is not surprising that the secondary evils of amputation should be so similar to those of the puerperal state." Cruvelhier has seized this analogy in its minutest details—and his words are quoted by our author.

"Whether, then, I turn to the analogies afforded by comparative anatomy; or to the direct evidence afforded by inspecting the human uterus, in a healthy state, soon after delivery; or whether I look to the authorities of competent anatomists,—I find, that after child-birth the womb is like an amputated stump, and that it has a reparative process to perform, which, being disturbed, permits the large gaping vessels to spread in the blood noxious secretions which they have imbibed." 80.

PROPOSITION III.

That the various forms of puerperal fever depend on the one cause of vitiated blood, seems to be the most difficult task of all—but it is grappled with manfully by our ingenious author. He sets out, indeed, by assuming that the experiments of Gaspard must have led every one of his readers to this inevitable conclusion, since in those experiments it was seen that the vitiated blood produced in one case one group of lesions, in another case a different group, and so on—the variation being not only of place or organ, but of intensity.

"But how, it will be asked, are we to account for this partitioning off of so diffuse a malady, as that induced by vitiation of the blood? why is it not always spread wherever there is blood? and why are puerperal fevers, now peritonitic alone, now metro-peritonitic, now gastro-enteric, and now falling on the nervous centres?

We know that the blood-vessels, like every other part of the body, are in their nature capable, of themselves, of repairing injury, and stemming the actions of disease. The experiments of Gaspard and Cruvelhier, permit us to infer that there is always an endeavour to hem in the noxious cause as near to the spot first injured as possible; and Mr. Arnott has remarked,—what Cruvelhier had stated in 1820—that coagula form, to prevent the spread of inflammation of a venous trunk; while the former gentleman, more minute in his investigation, has always found that the injured vessel is obliterated only up to the first branch it gives off: as if nature, while endeavouring to pen up the exciting cause of malady in as small a compass as possible, wished to use whatever she could of the other channels of circulation.

It is from this law, that we find the injuries of puerperal fever so often confined to the uterus and its appendages, to the lower portion of the peritoneum, and to the adjoining intestinal canal; for we have seen that the point of departure for the noxious matter, is in this disease from the veins of the uterus." 83.

As for the attempt to trace the paths by which distant organs are affected, he thinks this could be done, provided we knew which of the uterine veins became the channel of infection. This being impossible we must be content with the same degree of knowledge which we possess with regard to other poisons affecting the circulation.

"In conclusion, then, I deduce the first, or peritoneal form of puerperal fever, from the action of the poison being more or less confined to this membrane.

The second form, or the gastro-enteric; from the action on the liver, the organ through which, as the experiments of Gaspard and Fontana, and the admission of all physiologists shew, most poisons received into the system endeavour to escape. Whether the mucous membranes of the intestines are affected directly by the vitiated fluids, or secondarily through the acrid secretions of the liver, or in both ways as I believe; the group of symptoms constituting my second form of puerperal fever remains the same.

The third, or nervous form, I conceive to result from an impression on the nervous centres, not necessarily inflammatory, though it sometimes leads to inflammation. John Hunter speaks of this condition of the nervous system, under the metaphorical expression of 'alarm.' The first impressions of the most virulent poisons, are very commonly accompanied with panic and extreme agitations. Fontana found this to be the case with dogs, after inoculation with the viper poison. The symptoms produced by the poison of cholera, of small-pox, and of vegetable miasmata, are in a certain number of cases so characterised. The impression on the nervous system, which is transitory in other cases, and soon veiled by the specific symptoms of some organ or organs labouring under the attack, remains in this as the permanent feature of the disorder; but violent perturbations of the nerves are, under such circumstances, speedily fatal. Where death does not take place from nervous perturbation alone, the membranes of the brain, and the cerebral substance, are found altered by the same process as seen in other parts.

The fourth, or complicated form, is the result of a poison not confined to certain structures, as the peritoneum or uterus, where its violence is pent up and exhausted, but diffused by the circulation over many organs, causing each to react after its own laws, and giving to the disease it produces a character of inextricable confusion, and almost hopeless fatality." 86.

Thus ends this important Section, for which, indeed, the reader was pretty well prepared by all that preceded it. No one can fail to doubt or to admire the ingenuity with which he has worked up all the facts and reasonings that could be made to bear on the question. So systematically has he proceeded that the whole looks more like a sober induction, than a startling hypothesis. The theory, too, is very seductive. We delight to trace numerous, and apparently opposite phenomena to one single cause, and feel a kind of pride in being able to do so. In the present case, however, there is one startling consideration bearing on the theory of our author—namely, that the Almighty must have ordained that half the human race, and the most amiable half, should be not merely once, but many times in their lives, in the same state of danger as a man with an amputated leg! This is a serious drawback on population, and seems to have been overlooked by Malthus and the political economists. Fortunately for the warrior and the puerperal female, the conditions which render amputation and child-birth dangerous, are comparatively rare. The investigation of these conditions is not less important than that of the unique cause of puerperal fever, the result of these conditions. This investigation is rather briefly treated of in a Chapter in which he examines the opinions of various authors respecting the nature of puerperal fever. These opinions we shall pass over, but notice the—

### PUERPERAL CONSTITUTION.

The Germans attribute the essence of the fever in question to the efforts of Nature to bring back the female constitution to the state in which it was antecedent to pregnancy and parturition. The changes during utero-gestation are slowly made—the return to the ordinary state after child-birth, is rapid —and thus the tendency to disease is increased. The high susceptibility of the nervous system, at such periods, facilitates morbific impressions. The disturbance of lactation plays its part also. Still this theory of the Germans is regarded by our author as very defective.

Dr. Hall has connected this fever with intestinal irritation, and this latter was found in three-fifths of our author's cases. But a careful scrutiny led Dr. F. to infer that the intestinal irritation was secondary to the metro-peritonitic attack—that, in many instances of genuine puerperal fever, no intestinal irritation existed at all—and lastly, that gastro-enteric fever itself does not exhibit the phenomena of puerperal fever. Our author has already shewn that one of the commonest results of vitiated blood is irritation of the hepatic and intestinal functions. This will account for the occasional complication of the intestinal affection with puerperal fever. Dr. F. incidentally alludes here to the danger of exhibiting drastic purgatives in the puerperal state, as they often bring on violent pain and metro-peritonitis.

*The Influence of Seasons* must be placed in the catalogue of predisposing causes. The coldest and dampest portions of the year are the most fatal, and *vice versâ*. This is very constant. Still these alternations of season cannot be the main cause—they can only be influential, since whole seasons pass without any fever of this kind. It has been observed that when the disease is epidemic here, it is generally so on the Continent.

*Hospital Air.* On this head there is abundant evidence. The disease is most fatal in such establishments, as they are now managed. "A lying-in-hospital should consist either of a series of cottages, or its spacious wards should contain very few patients." Such a desideratum, however, is almost hopeless, for very obvious reasons.

"With regard to the General Lying-in Hospital,—its locality, rather below the level of the river, and surrounded by a mesh-work of open sewers, fifteen hundred feet in extent, receiving the filth of Lambeth, and some not thirty feet from the wards of the institution, may account for it unhealthiness. It is only after repeated remonstrances that these sources of pollution have, in part, now begun to be obliterated. In the absence of a medical police, nothing but a catastrophe, known under the gloss of a 'strong case,' has the slightest chance of remedy. Public bodies, like the commissioners of sewers, are hampered by their rigid customs, and by the penalties of the law, from coming forward, while individuals have little inclination, and less influence, in making the appeal." 104.

*Uterine Injury.* It has already been observed that contaminating matters may be introduced into the blood in two ways; by means of the secretions from the uterine wound, or by means of any injury to one or more of the uterine sinuses. The former source is considered to be analogous to the direct insertion of virus into healthy vessels, as after the viper-bite, dissection-wounds, &c. In the second case, "the source of injury is precisely similar to that arising from venesection, where the solid walls of the vein first inflame, and the matter exuded, is afterwards circulated."

"We should expect, *à priori,* that forcible disruption of the uterine veins by manual separation of the placenta—or that long-continued action of the uterus, compressing and irritating the large uterine wound, as in strong and long-continued after-pains,—that instrumental operations, and all such causes as directly bruise or disturb the wound,—would create diseased secretions from the traumatic surface, or irritate its large pendulous lacerated vessels, and so give rise to the phenomena of puerperal fever. These deductions, *à priori,* appear confirmed by facts.

It is well known that the first labour is generally much longer than any subsequent one; consequently we may assume that in first labours there is more mechanical injury to the uterus than in any other, and, therefore, a more unfavourable state for the healing of the traumatic uterine surface. Out of two hundred and four cases of our puerperal fever tables, more than eighty were first labours.

Again, of the total number of deaths, (amounting to sixty-eight) one-half were patients confined for the first time. These facts prove that the severity of a first labour, or mechanical injury, is a strong predisponent to puerperal disease. Of four hundred and fifty-six cases, Dugés finds that one-third more first labours than second are attacked; and Campbell, that of eighty-five attacked, twenty-nine were primiparæ.

After instrumental labours, in which mechanical injury must necessarily be inflicted, thirty-two cases occurred.

Now, as on an average artificial aid is not required more than once in fifty cases, the fact of thirty-two women being seized after instrumental delivery out of four hundred and fifty-six, clearly shews the influence of mechanical injury in producing puerperal fever. In these cases the injury is not the direct effect of the instruments, for that is known to be very slight and mostly none—but of that state of things which necessitates artificial aid." 107.

Dead children—abortions, hæmorrhages—depressing passions of the mind—insufficient food—suppression of the lochia, &c. may all prove exciting or predisposing causes of puerperal fever.

"I have, in conclusion, to protest against this my attempt being considered as a revival of the follies and errors of humoral pathologists with their four fluid constituents of blood, phlegm, bile, and atrabile; and their cosmic elements, fire, water, earth, and air, their occult causes, and their facile explanations. It has taken nearly 3000 years to convince physiologists that the whole of a living body is alive, and consequently subject to all the impressions and re-actions of the vital power. At first the fluids were the sole seat of life, and then the solids became exclusively gifted; and each hypothesis furnished the root of a branching nosological tree. Latterly, the best modern observers have traced much of disease and morbid formation to disorder in the fluids." 111.

Dr. Ferguson candidly admits that "what he has done he does not consider as new, but looks on it as an attempt to demonstrate what has been hitherto a matter of pure conjecture, and mere opinion." "He has availed himself of the valuable hints and developments in various sources of information, and has endeavoured to probe the views and compare the facts with each other and with his own." He has thus endeavoured to arrive at that great desideratum—"a just theory of this most fatal and most complex malady." In this state then we shall leave the theory in question, in order that it may be put to the test of time and experience. This brings us to the most important chapter of all.

### CHAP. V.—TREATMENT.

A statistical or numerical investigation of the various epidemics, in various localities and institutions, gives the melancholy result of one death in every three cases of puerperal fever.

### I. PERITONEAL FORM.

Whenever peritoneal pain was evinced, a larger linseed poultice over the abdomen was found beneficial in the Lying-in Hospital, often affording decided and powerful relief.

It should be so thick as to retain warmth for four hours—and sufficiently large to reach from the sternum to the pubes. Where there is obvious indication for venesection, ten grains of Dover's powder may be given when the poultice is applied. A second visit should be made in four hours, when, if the symptoms have been alleviated, a fresh poultice and another powder may be prescribed. If, in four hours, after the second medication, the practitioner is not satisfied that the malady is yielding, depletion must be resorted to at once. The powder may be combined with mercury and aperients, according to circumstances. Numerous cases are detailed, some fatal, others successful. We shall give one as a sample.

"*Case* XX.—Charlotte Crisby, ætat. 18, February 12, 1829. 13th. On the evening of the first day, after a tedious labour, abdominal pain, extending from the umbilicus downwards, attacked her; the pulse was constant, increased by pressure; the pulse full; the whole appearance that of a plethoric person; venæsectio ad deliq., about ℥xx were taken away, she did not faint, but the pulse at the wrist ceased for ten minutes. The blood was flat, sizy, and not at all buffed. The crassamentum was firmish; grs. x, Dover's powder every four hours.

14th. In twelve hours from her attack she was free from all pain, but after a few hours it returned in the right groin, extending all over the belly, up to the very pit of the stomach. It was increased by the slightest pressure, and was attended with painful paroxysms recurring every half hour. Milk and lochia copious; head-ache; pulse 130, feeble: twenty-four leeches. In six hours after this all pain had subsided. Two grs. calomel every two hours; fomentation and poultices.

15th. There was a little soreness; the gums were just touched, otherwise well.

16th. Gums still sore, but no pain any where; well. The milk and lochia natural throughout. MR. CATHCART.

*Remarks.*—This is an example of the use of mercury, combined with venesection; it is highly instructive." 147.

The following passage we deem it right to extract.

"The following is the sum of my own experience in bleeding as a remedy in puerperal fever:—Of all the means we possess of arresting this malady, I believe bleeding general or topical to be by far the most extensively applicable. The cases in which it is not so, are exceptions to the rule. Mercury, turpentine, emetics, opiates, sudorifics, &c., have a more limited range of utility than abstraction of blood. But while I admit this, I am equally certain, that *large* bleeding has not been borne in this malady, generally speaking, during the last twelve years.

Those who have borne it best and required it most, were—1. Those who were originally vigorous, and in whom no chronic ailment of the intestinal canal or lungs previously existed. 2. Those in whom the fever was accompanied by a general turgor of the frame: their aspect being that of a person who has been flushed by running, and forming a marked contrast with the pinched, shrivelled, and stricken looks of those labouring under the typhoid form of the malady. 3. Those in whom the disease seemed to be limited to one organ.

It may be asserted, with more hesitation, however, that they who are confined out of a hospital, exhibit greater reactive powers than those who are confined in one.

The pulse, as Gordon has remarked, is very deceptive; and the cases I have given, shew that painfulness is no sufficient criterion of the necessity of depletion.

Besides these general indications, epidemic puerperal fever, has, invariably, the character common to the ordinary fevers raging with it: if the latter require depletions, the presumption is, that the former will also.

It is curious, that in the majority of cases bled, the blood is neither cupped nor buffed.

The persons who do not bear large bleedings are those attacked by the ataxic or gastro-enteric forms: even though they be of originally strong constitutions: also those labouring under the complicated form, where many organs are, simultaneously, under the grasp of the diffusive malady. But in this last class there are so many shades of disease, that no absolute rule can be laid down.

If large bleeding be determined on, it must, to be beneficial, be resorted to, within the first twenty-four hours of the attack. In the second stage of the disease it is often, rapidly, fatal. If the bleeding be made early, it may be often repeated. It appears, where it does not remove the malady, to stop its progress, and make it continue lingering in its first stage, so that the repetition of venesection is late, only as to the lapse of time, but not tardy, as to the progress of the disease.

Of local depletion by leeching, it may be said, that the cases in all the four forms of puerperal fever, are very few indeed, which do not permit us to resort to it. It often removes a pain which will not yield to blood-letting. The number of leeches required will, of course, vary with the case; in some, six is sufficient; in others, six dozen will scarcely be so. But the average cases will require from two to three dozen." 154.

### II. GASTRO-ENTERIC FORM.

In this form the local inflammations are slight and transient, or non-existent, whilst the general symptoms resemble typhus accompanied by intestinal irritation. Dr. F. considers the disordered secretions the result of vitiated blood, and not the cause of that vitiation. In this respect he differs from Dr. M. Hall, who attributes the disease to scybala and loaded bowels.

"For some of these cases which are not accounted for from the irritation of loaded intestine, Dr. M. Hall is obliged to assume the effects of loss of blood. But these effects, which that eminent pathologist has most ably traced to the impression made on the nervous functions, take place in this form of puerperal fever where there has been no hæmorrhage." 156.

Dr. F. agrees, however, with Dr. Hall as to the treatment of the malady. He thinks active purgatives inadmissible—and when an epidemic breaks out in the hospital, the usual routine of giving the "black-dose" on the third day after parturition, is abandoned. Where accumulations are suspected, castor oil is the safest aperient, guarded by hyosciamus or hop. General bleeding is rarely necessary. The topical inflammation is best relieved by leeches—and even these sometimes cause fainting. The nervous system is greatly affected in this form of fever, and the constitution soon pulled down. The strength is therefore to be economized.

"The following I have found the most suitable treatment. Get rid of all local inflammations as soon as possible, by leeching or by moderate depletion, so as to reduce the malady into simple fever, with gastro-enteric irritation. When the skin is early dusky, and there is nausea or vomiting, begin with an emetic. If there be no nausea, nor vomiting, but intestinal flux, with a red tongue smeared with suburra, a large dose of calomel, from ten to fifteen grs. should be given. Small doses create purging, pain, and irritation, while the full dose produces one to six large pultaceous stools, after which the tongue is cleaned, rendered less red, and more moist, and the pulse usually falls. These stools, when examined, appear to contain the fæcal matter suspended in large quantities of mucus and greenish bile, as if the turgid capillaries of the irritated intestinal canal and liver had been freed from their load. In some instances, a repetition only, of the same dose is required to efface the main features of the malady, and to leave nothing but debility to support. In others, after a short respite, diarrhœa recommences, and soon is apt to become colliquative.

For this state, when the secretions are diseased, as well as copious, a treatment essentially alterative should be used, as a combination of Dover's powder, with a mercurial. When, however, the chief danger arises from the frequency and quantity of the flux, it will be requisite to resort to absorbents and astringents. If the debility be great, wine may be freely given with gruel or sago; where it is not, the strength should be supported by soup, *thickened* with any gelatinous substance, as thin fluids almost always cause immediate purging. At night, when delirium and night-mare, and fantastic visions torment the patient, a full dose of Batleys's sedative, in camphor mixture, strengthened by a few additional grains of camphor, should be given. After the intestinal irritation has abated, tonics may be used, and these are always better borne when ammonia is a chief ingredient." 160.

Several cases are detailed, and comments made by the author, for which we must refer to the book,

### III. Treatment of the Ataxic Form.

Great disturbance of function will kill more rapidly than organic changes. The mysteries of the nervous system are yet locked up in Cymmerian darkness! Our author, and every practitioner, have seen death take place in this and other diseases, without any trace being left in the dead body to account for the fatal event. This state was designated by John Hunter—"action without power"—it is evidently debility combined with irritability.

"Our practice, then, in the ataxic form of puerperal fever, if it be, as I believe, a malady to which the term 'action without power' is singularly applicable, is to sustain; where there is sinking, the support must be by stimuli, largely and frequently given. Where there is no visible sinking, then stimuli are not indicated, but sedatives, especially the Batley's laudanum, should be immediately given. When the action is equalized and reduced, nourishing food and tonics may be administered." 179.

Cases and remarks follow, with extracts from various authors.

### IV.—Treatment of the Complicated Form.

It is only in its slighter grades that this form is curable. "Where it is the leading characteristic of an epidemic, the vast majority will die." Fortunately this most fatal kind of puerperal fever rarely constitutes the whole of an epidemic.

"What treatment," says Cruvelhier,* "shall we oppose to purulent infection? To this question experience is as yet dumb, while theory would seem to point to diffusible stimuli and tonics; to ammonia, quinine, and to sudorifics; to hot external applications; to the vapour-bath; to purgatives, especially to emetics; to tartarized antimony, in large doses; to vesicatories, and to strong diuretics. Calomel has been extensively employed to create a fluxion from the intestinal mucous membrane; but all these means have failed as signally in my hands as in those of others. Yet when the injection of putrid matters into the veins of living animals has been followed by abundant, and very fetid evacuations, they have usually got well. It is a fundamental fact of pathology, that the intestinal canal is chiefly affected in diseases caused by any miasmata. The ancients expressed this truth by saying, that the intestinal canal attracted the poison of febrile diseases. I am certain that diseases, resulting from purulent infection, would not be stamped with the seal of incurability—and that nature, seconded by art, would triumph in the majority of cases—if the pus, which is incessantly renewed, did not incessantly renew the sources of infection." 200.

Dr. F. then enquires if there be no landmarks to guide us in discriminating between the fitness of various remedies, as mercury, quinine, emetics, stimuli, turpentine, sudorifics, blisters, diuretics, &c.? He then discusses the merits of each of these remedies allotting to them their due value, and endeavouring to ascertain the circumstances in which they may be advantageously or injuriously administered.

---

* "Art. Phlebite, Dict. de Med. et Chirurg. pratiques."

1. *Emetics*. These, he thinks, are most beneficial when the violence of the disease falls on the liver, and when there is early nausea and spontaneous vomiting.

2. *Purgatives*. " My own experience with regard to aperients is, that whenever they create tormina, there is the greatest risk of an attack of metro-peritonitis succeeding. This so constantly occurs, that I invariably mix some anodyne—usually Dover's powder, or hyoscyamus, or hop, with the purgative." 211.

3. *Mercurials*. Here our author introduces a long dissertation on the action of mercury, derived chiefly from certain lectures delivered by Dr. Farre, and which, amongst some questionable speculations, are to be found many judicious remarks. The continental physicians, as Dr. Ferguson observes, have scarcely any just notions of the physiological effects of mercury on the human system.

" ' Mercurial action,' says Dr. Farre, ' is positively anti-phlegmonous. If it be pushed far enough, it produces an effect the exact reverse of this phlegmonous state, namely, the erythematous inflammation; the tendency of which is to loosen texture, while that of phlegmonous inflammation is to bind texture.' " 215.

Besides this, mercury opens and augments all the secretions and excretions, and thus reduces the whole bulk of solids and fluids. There is a period in most inflammations when we are obliged to desist from the lancet without having subdued the disease. Here mercury is an invaluable agent. The following are Dr. Farre's dogmas respecting mercury.

" ' 1. Never to give mercury where there is an idiosyncracy against it.' The following case is illustrative of the danger of neglecting this advice :—

' A patient of Mr. G.'s, of the Borough, desired him never to give her any mercury, as that drug was a poison to her whole family, to which he, without arguing the point, at once assented. In Mr. G.'s absence, the late Mr. C. was consulted as to some trifling disorder of the bowels, and not knowing the peculiarity of his patient's constitution, prescribed two grains of calomel. The next morning the lady shewed the prescription to Mr. C., saying that she was sure she had taken mercury, as she felt it in her mouth. In a few hours ptyalism ensued ; in consequence of which she lost her teeth, her jaw exfoliated, and she ultimately, after a succession of ailments, died, in about two years.'

' 2. Mercury should be used in all active congestions—Pyrexia, phlogosis, phlegmon, ophthalmia, strabismus, cynanche laryngea, cynanche trachealis, pneumonia, and in all inflammatory diseases. In the adhesive stages of dysentery, in the phlegmasiæ, where there is inflammation with power, in tetanus, hemiplegia, paraplegia, neuralgia, in their states of active congestion.

' 3. Mercury is hurtful, or doubtful—In the malignant or asthenic forms of pyrexia, where there is low delirium ; but in phrenitis, and in that peculiar form of it, the ' coup de soleil,' it is most effectual. It is hurtful in tetanus from punctured wound, and in all cases of irritable disease.

' In idiopathic iritis, it is as effectual as bark in ague ; but in the traumatic it is injurious, as it interferes with the closing of the vessels by adhesive inflammation : hence in all hæmorrhage, where the orifices of vessels require to be closed, it is hurtful.

' In the hemiplegia of lesion, in asthenic paraplegia, in the neuralgia of irritation, it is bad. Poor Pemberton was three times salivated for tic douloureux, and three times the worse for it.

'It is hurtful in the inveterate forms of scrofulous ophthalmia, though useful in the early stage. It is bad in the amaurosis of depletion.

'It is useful in puerperal peritonitis, and hurtful in the typhoid form of it; as also in the ulcerative stage of dysentery.

'In general, it is doubtful in the suppurative stages of inflammation, and in all erysipelatous and erythematous inflammations, or those tending to gangrene. It is hurtful in all cases of pure asthenia from deficiency of red blood.'" 222.

The oxymuriate is sometimes preferred to calomel.

*Turpentine.*—Dr. F. has no experience of this remedy. Most patients have an unconquerable loathing of it, and indeed we wonder how any puerperal woman can get it down or keep it down.

*Vesicatories and Diuretics* are useful auxiliaries—the former after proper depletion. Dr. F. has tried Dr. Stevens's celebrated mixture, without effect of any kind.

"There still remain a few points of interest which I will very briefly discuss.—

In the complicated form of puerperal fever, the 'deposits' which take place in the limbs and eye, require much attention in the treatment. If there be constitutional vigour, or if the part be affected early in the malady, leeches may be applied. They are contra-indicated when these disorganising processes appear in frames enfeebled by disease or constitutional causes. Two or three examples are recorded in my tables, where a few leeches applied, late in the disease, caused immediate sinking. The local inflammation is, even in the very last moments of life, exceedingly painful, and seems to demand depletion; but, unless the whole state of the patient be taken into the account, a dozen of leeches will turn the vibrating scale from life to death.

It is when the eye is attacked that leeching will be oftenest useful. When the seat of deposit is in the cellular and muscular portions of the limb, it should be covered either with a linseed-meal poultice, or with flannels soaked in decoction of poppy and camomile flowers. The ease obtained is very great; the swelling subsides in many instances entirely, leaving the limb unscathed; in others it is removed in every part but two or three spots which are found to be puffed out with pus, which should be evacuated. Where deposition takes place in a joint, the treatment by leeching and poulticing, will sometimes arrest its disorganisation: in all it will give ease. But there is a tardy convalescence to be looked for, and, even with the best surgical attention, it is often impossible to prevent the loss of motion of the affected limb.

There is another effect of this fearful malady, which I have remarked, but never seen described. Persons who have recovered from an attack of puerperal fever, apparently of no great urgency, often do not regain health for several months, nay, even for one or two years. Their pulse continues rapid and irritable, and scarcely an evening passes without slight febrile excitement. In some, boils or abscesses break out from time to time; in others, the mucous membrane of the intestinal canal is affected by the presence of a painful spot, or by great irritability, and the consequent variation in the quantity and quality of its secretions. In all there is much emaciation.

This state of constitution is often produced after exanthematous fevers, and I have known it occur in two instances after puncture from dissection. As yet I have seen no fatal termination to this very distressing, and, to the friends of the patient, and the patient herself, alarming state of things. The plan pursued by me, in its treatment, has been,—1. A sustained course of sarsaparilla and mercurial alteratives. 2. The warm bath twice a-week. 3. A change of climate and the use of some of the foreign mineral waters, selected with reference to the peculiarities of the case.

Besides this insidious state of chronic disorder, there is recorded, by most authors, a more obvious derangement of health, the consequences of the effusion into the peritoneal cavity. In these cases the patient either sinks from hectic, or the effused fluid finds a vent through the abdominal parietes. I have, in some five or six cases, remarked the following coincidence, viz.,—The subsidence of a tumid abdomen, and the evacuation, through the vagina, of sero-purulent fluid, in such quantity, as to give the patient and nurse the notion of the bursting of an internal abscess. On examination I could detect no uterine lesion and no perforation.

Could the fluid, effused into the peritoneal cavity, have escaped through the fallopian tubes into the uterus, and thence into the vagina?" 228.

A considerable number of dissections follow, and several valuable statistical tables close the work—a publication which few practitioners will fail to peruse and study, after the full and careful analysis which we have presented them. The ingenious theory which our author has adopted rather than invented, cannot fail to excite one reflection—and that not a very consolatory one, in every thinking mind. Over the primary, the essential cause of puerperal fever, we have no control! We cannot prevent the contaminating matter or miasm from draining into the blood—nor can we neutralize or decompose it when circulating through the body. All we can do is to attempt to counteract the *effects* which the poison produces on different tissues and organs of the body—but without knowing any specific remedies for such counteraction! We know, to our cost, that the disturbances and diseases roused into action by the febrific miasm of puerperal fever, whatever that miasm may be, are infinitely more unmanageable, and more dangerous in their results, than inflammatory or other lesions arising from other causes. Thus the practitioner is surrounded with doubts and difficulties on every side, and the theory here advocated is unequal to the task of extricating us from the labyrinth—even by the ingenious clue which it furnishes. It is always best, however, to know the TRUTH, whether that truth disclose or not the remedy for the evil. In taking leave of our highly-gifted author, we tender him our best thanks for the pleasure and information which we reaped from a perusal of his volume. Perge pede quo cepisti.

